# Use of *Euphorbia balsamifera* Extract in Precursor Fabrication of Silver Nanoparticles for Efficient Removal of Bromocresol Green and Bromophenol Blue Toxic Dyes

**DOI:** 10.3390/molecules28093934

**Published:** 2023-05-06

**Authors:** Salha M. Aljubiri, Walaa H. El-Shwiniy, Ayman A. O. Younes, Eid H. Alosaimi, Badr Abd El-wahaab

**Affiliations:** 1 Department of Chemistry, College of Science, University of Bisha, P.O. Box 511, Bisha 61922, Saudi Arabia; 2 Faculty of Science, Chemistry Department, Zagazig University, Zagazig 44519, Egypt; badr.hassan@zu.edu.eg

**Keywords:** Ag-NPs, *Euphorbia balsamifera*, bromocresol green, bromophenol blue, decolorization

## Abstract

Silver nanoparticles (Ag-NPs) are attracting great attention for their use in various applications, along with methods for their green and facile production. In this study, we present a new eco-friendly approach based on the use of *Euphorbia balsamifera* extract (EBE) in the green synthesis of silver nanoparticles (Ag-NPs), which are then applied as a reducing and stabilizing agent for the efficient removal of water-based reactive dyes such as bromocresol green (BCG) and bromophenol blue (BPB). The as-prepared Ag-NPs are quasi-spherical in shape, with an average diameter of 20–34 nm. Diverse characterization methods, including X-ray diffractometry (XRD), Fourier transform infrared spectroscopy (FT-IR), scanning electron microscopy (SEM), transmission electron microscopy (TEM), and Brunauer–Emmett–Teller (BET) analysis, were used to analyze these Ag-NPs. The results reveal that water-soluble biomolecules in the *Euphorbia balsamifera* extract play an important role in the formation of the Ag-NPs. The removal of toxic dyes was studied under varied operational parameters such as Ag-NP dosage, initial dye concentration, pH, stirring time, and temperature. Under the optimum investigated conditions, nearly 99.12% and 97.25% of the bromocresol green and bromophenol blue dyes, respectively, were removed. Both BCG and BPB adsorption were found to adhere to pseudo-second-order kinetics (r_2_^2^ = 1 and 0.995) and fit the Langmuir isotherm models well (R_1_^2^ = 0.998 and 0.994), with maximal monolayer adsorption capacities of 20.40 and 41.03 mg/g, respectively. Their adsorption processes were observed to be intrinsically endothermic. The results confirm the potential of the *Euphorbia balsamifera* extract as a low-cost, nontoxic, and eco-friendly natural resource for the synthesis of Ag-NPs that may be useful in the remediation of hazardous dye-contaminated water sources.

## 1. Introduction

**Figure 1:**
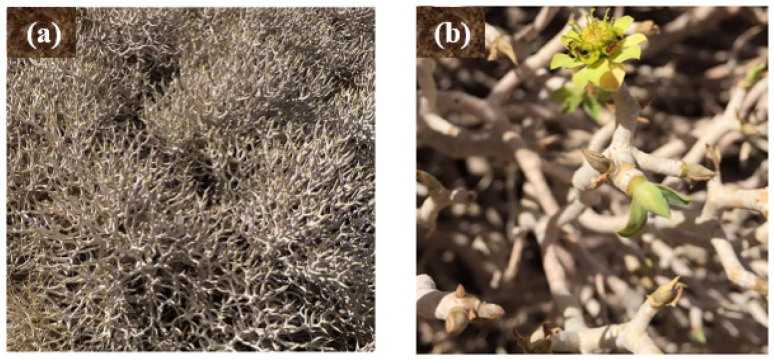
Optical images with (**a**) low and (**b**) high magnifications of *E. balsamifera* from the Asir region.

The water pollution and health risks associated with dye contamination are major concerns in practically all developing nations due to the use of dyes in numerous industries for adorning and preserving various materials, including through the coloring of textiles, leather, paper, and other materials [1]. All dye effluent from the manufacturing and finishing of textiles is discarded into waterways, resulting in these waterways becoming filled with a variety of toxic organic substances that are harmful to people, as well as fish and other aquatic life [2]. Therefore, it is crucial to eliminate hazardous dyes from the water [3]. A variety of methods have been utilized to remove dyes from water, such as adsorption [4], electrochemical oxidation [5], ion exchange [6], and other techniques involving nanoparticles [7,8,9,10,11,12]. Nowadays, nanobiotechnology is of significant importance due to its widespread applicability in water treatment [13,14,15]. In particular, the green synthesis of plant extracts appears increasingly more attractive because plants can gather specific amounts of heavy metals in their various sections. Consequently, biosynthetic approaches utilizing plant extracts are gaining popularity as quick, easy, affordable, and practical ways to produce nanoparticles, in some cases serving as an effective substitute for conventional manufacturing processes [16]. Numerous plants can be employed to stabilize the reduction in the reaction-based synthesis of metal nanoparticles [17]. Many researchers have used green synthetic methods to produce metal/metal oxide nanoparticles from plant extracts and further investigated their different applications [18,19,20]. As traditional chemical procedures consume greater amounts of energy and reagents (which, in addition, are occasionally dangerous and toxic) compared with biological approaches, the green synthesis of metallic NPs is growing in importance as a topic of study for multidisciplinary scientists throughout the world [21]. Plants contain biomolecules that have a high potential for converting metal salts into nanoparticles. Plant extract-assisted production of silver and gold metal nanoparticles was first explored using extracts of aloe vera, oat, alfalfa, lemon, and neem [16]. The green synthesis of silver nanoparticles (Ag-NPs), which utilizes a variety of microorganisms and plants, represents a natural, biocompatible, and environmentally safe method [22]. Recently, the production of Ag-NPs utilizing plant extracts as reducing agents has been investigated [23,24]. Secondary metabolites, such as flavonoids, tannins, saponins, phenolic compounds, and protein, in plant extracts are key to the synthesis of Ag-NPs based on silver ion reduction [19]. Previous research has shown that the bioreduction mechanism can be broken down into three key steps: reduction and nucleation of silver ions, development and aggregation, and the final stage of capping and stabilization [25]. In most cases, plant phytochemicals play a decisive role as both capping agents and stabilizers [26]. The present study is focused on the green synthesis of Ag-NPs using *Euphorbia balsamifera*. It is a member of the Euphorbiaceae family, which is one of the biggest families, with over 330 genera and 8000 species, and it is characterized by a wide range of therapeutic properties [27,28]. *E. balsamifera* occurs in the Canary Islands, southwestern Morocco, Mauritania, western Niger, Sudan, Somalia, southwestern Oman, Yemen, and Saudi Arabia [29]. It is a pachycaul, dioecious, succulent dendroid shrub that may grow on rocky to sandy substrates and is adaptable to harsh temperature environments and characterized by its milky latex (Figure 1). The results from phytochemical screening of *E. balsamifera* extracts demonstrate the presence of terpenoid, steroid, tannins, flavonoids, cardiac glycosides, and saponins [30,31].

To the best of our knowledge and based on a review of the literature, no research has been performed on the synthesis of metal nanoparticles using *E. balsamifera*. Therefore, in this study, Ag-NPs were synthesized using *E. balsamifera*, and their efficiency in removing toxic dyes in water treatment was evaluated. The synthesized Ag-NPs were successfully applied in the removal of the bromocresol green (BCG) and bromophenol blue (BPB) dyes from water, for which they achieved a high percentage. In addition, different operational conditions affecting the water treatment process, such as Ag-NP dosage, initial dye concentration, pH, stirring time, and temperature were investigated.

## 2. Results and Discussion

### 2.1. Characterization of Silver Nanoparticles

#### 2.1.1. Powder X-ray Diffraction Studies (XRD)

**Figure 2:**
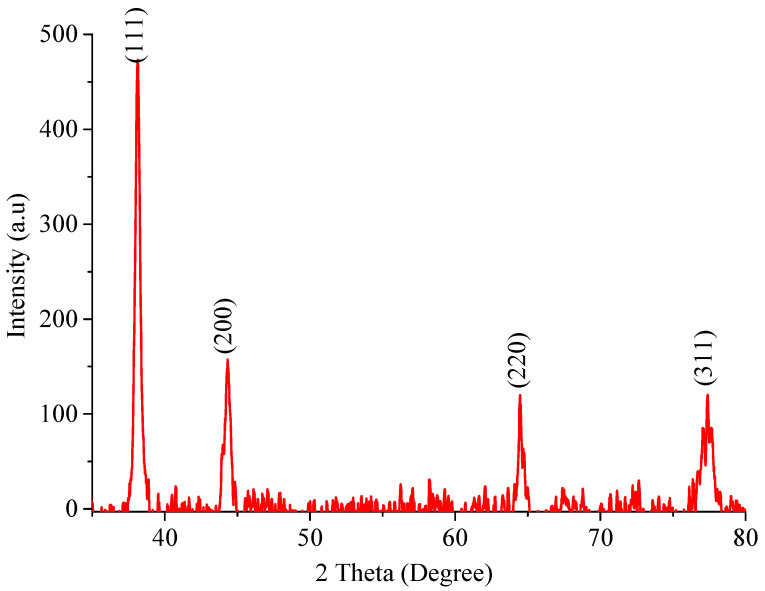
The Ag-NP XRD pattern.

The X-ray diffraction pattern of the prepared silver nanoparticles was recorded in the range of 30 ˂ 2θ ˂ 80, as shown in Figure 2. In the experimental diffractogram, the appearance of four 2θ peaks at 38.048°, 44.3228°, 64.6967°, and 77.3552° is attributed to silver metal and could, respectively, be assigned based on (hkl) principles as (111), (200), (220), and (311) crystalline structures of the face-centered cubic (FCC)-fabricated nanosilver. These four peaks were compared and matched with the standard powder diffraction card of the Joint Committee on Powder Diffraction Standards (JCPDS), silver file No. 04-0783 [32]. The XRD data demonstrate the crystalline character of the generated Ag-NPs. The mean crystallite size t of the Ag-NPs was calculated to be 9.9692–19.1210 nm, as shown in Table 1. This calculation was performed using the standard Debye–Scherrer equation—t= kλ/β. cosθ, where t is the crystallite size in nm, k is a constant dependent on crystallite shape equal to 0.89, λ is the X-ray wavelength equal to 0.1542 nm, θ is the diffraction angle in degrees, and β is the full diffraction peak width at half maximum intensity (FWHM) in radians. The results of XRD analysis demonstrate that a reduction in silver ions by EBE is a feasible method for producing Ag-NPs that are structurally characterized by well-defined edges.

#### 2.1.2. IR, TGA, and Ultraviolet/Visible Absorption Spectra

**Figure 3:**
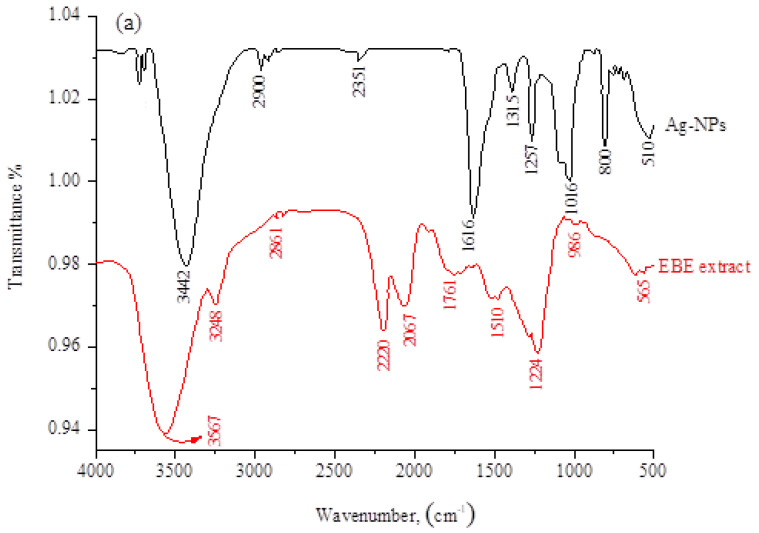

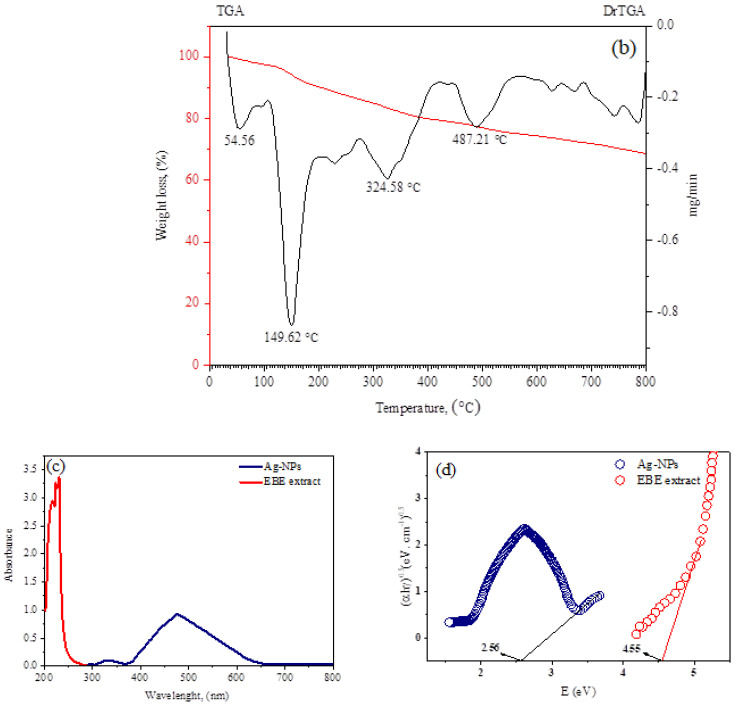
(**a**) IR spectra, (**b**) TGA, (**c**) UV spectra, and (**d**) band gap plot of Ag−NPs and EBE.

The biomolecules bound to the synthesized Ag-NPs via specific functional groups were identified by FT-IR spectroscopy. Figure 3a shows the FT-IR spectra of EBE and Ag-NPs. For the EBE, the broad signal at 3567 cm^−1^ (shifted to 3442 cm^−1^ in Ag-NPs) is attributed to the stretching vibration of O–H bonds [33]. The peak at 2861 cm^−1^ is attributed to the asymmetric stretching of the C–H bonds of alkanes and is shifted to a higher frequency (2900 cm^−1^) in Ag-NPs, when compared with the extract. The signal at 1761 cm^−1^ (is absent in Ag-NPs due to the reduction process) is attributed to the stretching vibrations of C=O in the amide bands [34]. The band 986 cm^−1^ is responsible for C-O-C stretching, which could be attributed to the reduction in Ag^+^ because the band was shifted to 1016 cm^−1^ in Ag-NPs [35]. The band 1510 cm^−1^ in extraction was due to the presence of amide vibrations, and this band was shifted to 1616 cm^−1^ in Ag-NPs because of the proteins that possibly bound to Ag-NPs through the amine groups. The stretching vibration of the C–N bond attributed to amines can be clearly observed at 1224 cm^−1^ in extract and absent in Ag-NPs [36]. In the Ag-NPs spectrum, the bands at 881 cm^−1^ are characteristic of out-of-plane C–H flexural vibrations, and those at 510 cm^−1^ confirm the existence of Ag-NPs. The above data suggest that the water-soluble biomolecules containing phenol, carboxyl, and amide groups, such as proteins or flavonoids, cap and stabilize the synthesized Ag-NPs through physical absorption rather than chemical bonds [37]. The thermal stability of adsorbent materials is important as it provides information on their behavior under various temperature conditions. Thus, we investigated the stability of Ag-NPS with temperature by applying thermo-gravimetric analysis (TGA), as shown in Figure 3b. TGA analysis was carried out in a nitrogen atmosphere at a heating rate of 10 °C/min at temperatures ranging from ambient temperature to 800 °C. Figure 3b depicts TGA curves of powder silver nanoparticles. From the thermogram, a slight weight loss of Ag-NPs was recorded from 40 to 250 °C, which is attributed to the removal of moisture and volatile components from the adsorbents. This was followed by significant weight loss from 250 to 450 °C, mainly due to the decomposition of organic matter. Ag-NPs were shown to be more stable for pollutant removal at high temperature conditions due to their lowered weight loss (total weight loss of 31.11%).

The production of Ag-NPs in solution was also verified using UV/visible spectroscopy. The solution was scanned in the wavelength range of 300–800 nm. Figure 3c depicts the characteristic pronounced peak absorbance of Ag-NPs at 470 nm, indicating the production of NPs of diverse architectures [38]. Absorption spectra are influenced by the particle size, dielectric medium, and chemical environment. The peak at about 330 nm is caused by unabsorbed biosubstances at the interface with Ag-NPs [39]. The Ag-NPs remained stable for three months and exhibited an average absorbance of 430–480 nm, which is within the range for Ag-NPs. This is evidence of the optical stability of the nanoparticles.

The optical band gap was estimated using Tauc’s equation αhv = (hv − E_g_)^n^, where hν is the photon energy, h is Planck’s constant, *n* is equal to 1/2 and 2 for direct and indirect transitions, respectively, and α is the absorption coefficient. A is an energy-independent constant. Plots of (νhα)^2^ and (νhα)^1/2^ versus *hv* were produced, in which a direct band gap was found by extrapolating the linear portion of the curve to (νhα)^1/2^ = 0, as seen in Figure 3d. The band gaps for the Ag-NPs and EBE were 2.56 and 4.56 eV, respectively. This result reveals that Ag^+^ minimizes the optical band gap values of EBE. The movement of electrons toward Ag^+^ is responsible for this decrease in the band gap. It is suggested that Ag^+^ increases the mobilization of the EBE electrons by accepting them in its shell, thus expanding the width of the localized levels in the resulting Ag-NPs, and in turn, the band gap is diminished. This result has many applications in optics, electronics, and energy-conversion devices. In reality, a tiny band gap indicates the molecule is more electroconductive due to facilitating electronic transitions between HOMO and LUMO energy states. The produced Ag-NPs can be employed as semiconductors, and the determined values of their optical characteristics are in the same range as those reported for highly effective photovoltaic materials.

#### 2.1.3. Scanning Electron Microscopy, EDAX, and BET Analyses

**Figure 4:**
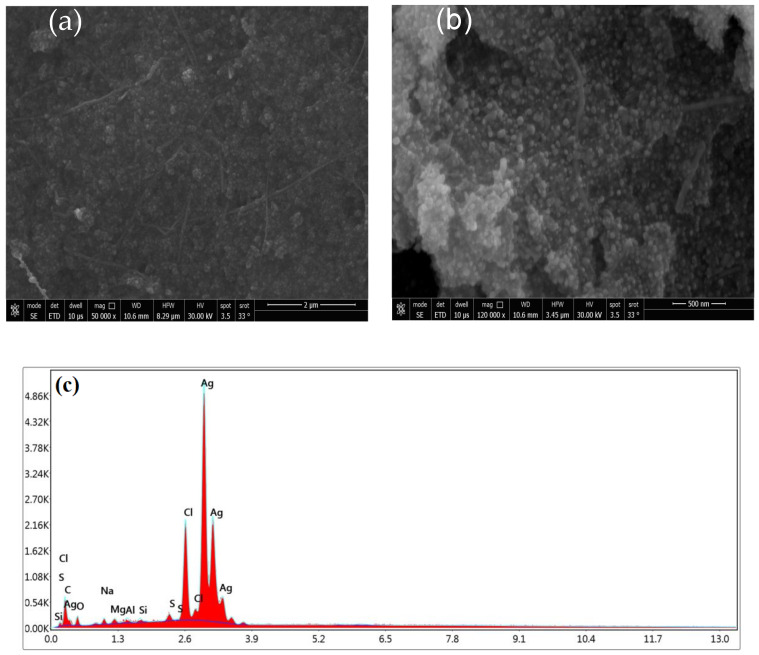
SEM images captured at (**a**) low and (**b**) high magnification, and the (**c**) EDAX spectrum of Ag-NPs.

The architecture of the as-prepared Ag-NPs was examined using SEM (Figure 4). The image captured at low magnification (Figure 4a) shows that the Ag-NP product mostly comprises agglomerates of irregular forms. The image captured at a high magnification, shown in (Figure 4b), reveals that these agglomerates are composed of spherical nanoparticles. It has been shown that the biosynthesis of Ag-NPs is regulated by a number of variables, such as time, metal salt, and concentration. In contrast, stabilizing agents and modifiers have proven to be crucial in controlling the shape of the granules by preventing aggregation [37,40,41]. Figure 4c depicts the elemental breakdown of Ag-NPs produced by green synthesis. Ag is viewed as the primary compound. Energy-dispersive X-ray spectroscopy (EDX) (Figure 4c) was utilized to record the elemental constituents of the Ag-NPs. A strong absorption peak located at 4.05 keV can be attributed to the elemental silver in the nanoparticles. In addition, the signals observed for carbon, oxygen, and sulfur confirm that the Ag-NPs were successfully capped by compounds from the EBE [37,42]. The Ag-NPs were measured using the Brunauer–Emmett–Teller (BET) method, with which their surface area and pore size distribution are precisely determined. The values of 2.20 nm, 0.464 cm^3^/g, and 618.736 m^2^/g were found for their mean pore diameter, pore volume, and specific surface area, respectively. The increase in surface area provides more contact and exposed areas for dye adsorption, which leads to a higher adsorption capacity.

**Figure 5:**
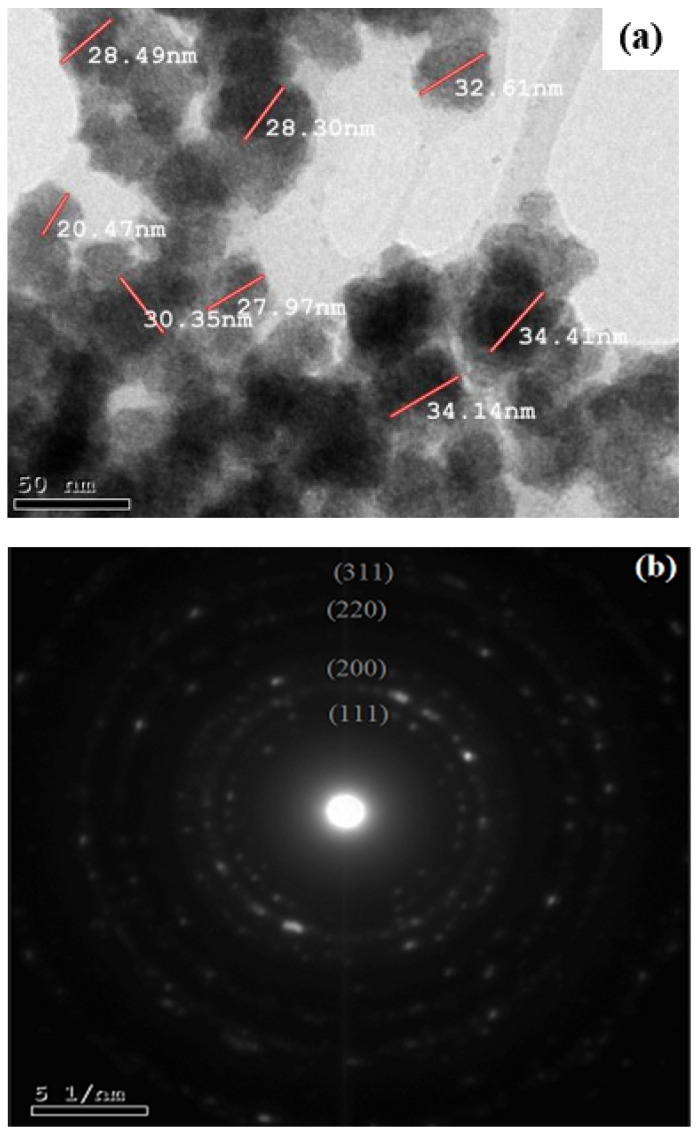
TEM image (**a**) and SAED pattern (**b**) of the Ag-NPs.

Figure 5a is a TEM image of the Ag-NP product, showing its quasi-circular architecture with a particle size of roughly 20–34 nm. In the SAED pattern (Figure 5b), five bright circular rings attributed to the (111), (200), (220), (311), and (222) faces are characteristic of the face-centered cubic crystals of silver. The XRD data also prove the crystalline nature of the Ag-NPs. Likewise, the TEM images and XRD spectrum indicate that the Ag-NPs prepared using EBE are crystalline in nature. The combination of the biomolecules in the extract is what prompts the formation of spherical NPs [43]. By contrasting the luminance of various particle components, such as face-centered cubic (FCC) metal nano-clusters, twinning, or the planar defect, the twinned nanoparticles could be identified. In the face-centered cubic lattice of noble metals, various crystal planes have been found to have distinct surface energies [43,44,45].

### 2.2. Features of Silver Nanoparticle Adsorption

#### 2.2.1. Calibration Curves of Dyes

**Figure 6:**
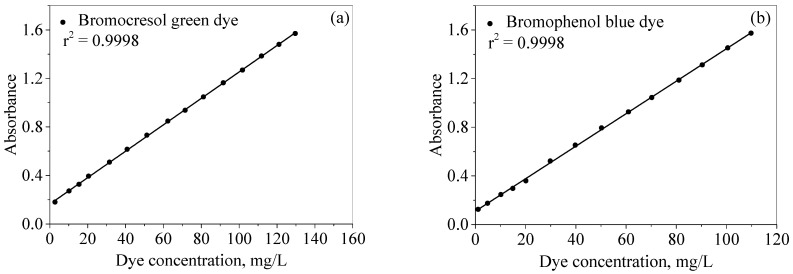
Calibration curves of (**a**) bromocresol green and (**b**) bromophenol blue dyes.

Dye concentrations were evaluated using UV/Vis spectrophotometry through the construction of a linear relationship between the dye concentrations and their UV/Vis absorbance (Beer–Lambert law). The calibration curves were prepared by measuring the absorbance of the dye concentrations (1.0–150.0 mg/L) at fixed wavelengths of 615 and 590 nm for the bromocresol green and bromophenol blue dyes, respectively. The dye concentration was then plotted against absorbance, as seen in Figure 6a,b. In the figures, it can be noted that linear relationships are present throughout the operating concentration ranges of 3.0–130.0 mg/L and 1.0–120.0 mg/L for the bromocresol green (BCG) and bromophenol blue (BPB) dyes, respectively. At higher concentrations, there is a breakdown of the Bee–Lambert law, resulting in a nonlinear relationship.

#### 2.2.2. Effect of Ag-NP Dosage

**Figure 7:**
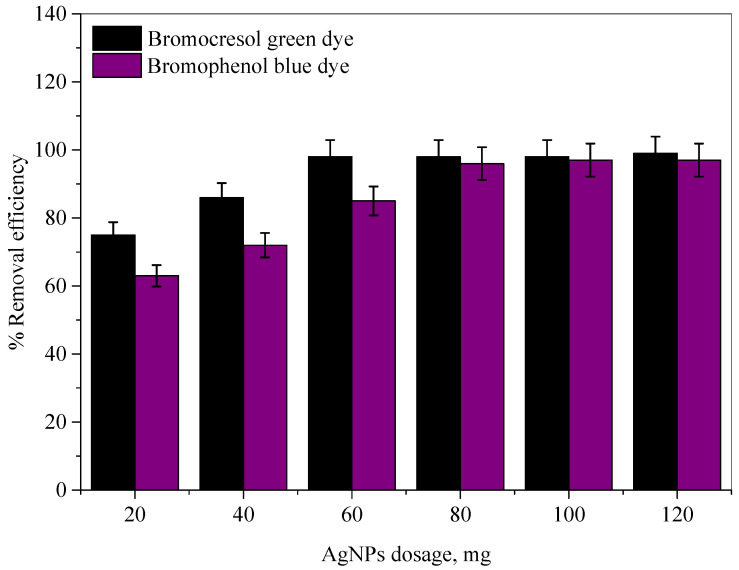
The effect of Ag-NP dosage on the color removal efficiencies of the bromocresol green and bromophenol blue dyes.

Ag-NP dosage is an essential parameter that influences the decolorization of the bromocresol green (BCG) and bromophenol blue (BPB) dyes because there exists an optimum dosage at which maximum dye decolorization occurs. In order to determine the optimum Ag-NP dosage, amounts of Ag-NPs ranging from 20 to 120 mg were added to 25 mL of each dye, with an initial concentration of 100 mg/L. A plot of Ag-NP dosage against the color removal efficiency of each dye is presented in Figure 7. It can be observed that the color removal efficiencies of the dyes increase with the increasing amount of Ag-NPs, starting from 10 mg and up to 60 mg for the BCG dye and up to 80 mg for the BPB dye. Thereafter, the color removal efficiencies remain consistent with the rise in the amount of Ag-NPs. The maximum color removal efficiencies were about 99.12% and 97.25%, with an optimum Ag-NP dosage of 60 and 80 mg for the BCG and BPB dyes, respectively.

#### 2.2.3. Effect of Initial Dye Concentration

**Figure 8:**
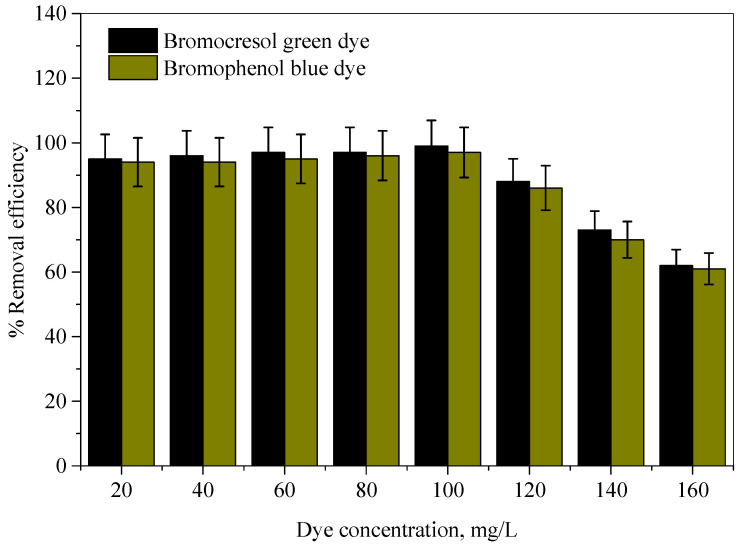
The color removal efficiency of Ag-NPs for different initial concentrations of the bromocresol green and bromophenol blue dyes.

The effect of initial dye concentration on the color removal efficiencies were examined over a wide range of BCG and BPB dye concentrations, from 20 to 160 mg/L for each, with the optimum Ag-NP dosage and solution volume kept constant at 25 mL. The results are displayed in Figure 8. This figure shows that the uptake of dyes was high at lower concentrations. It can also be seen that, at an initial concentration of 100 mg/L of each dye, the Ag-NPs removed about 99.12% and 97.25% of the BCG and BPB dyes by using the optimum Ag-NP dosage of 60 and 80 mg for the BCG and BPB dyes, respectively. With the further increase in initial dye concentration, the color removal efficiencies decreased.

#### 2.2.4. Effect of pH

**Figure 9:**
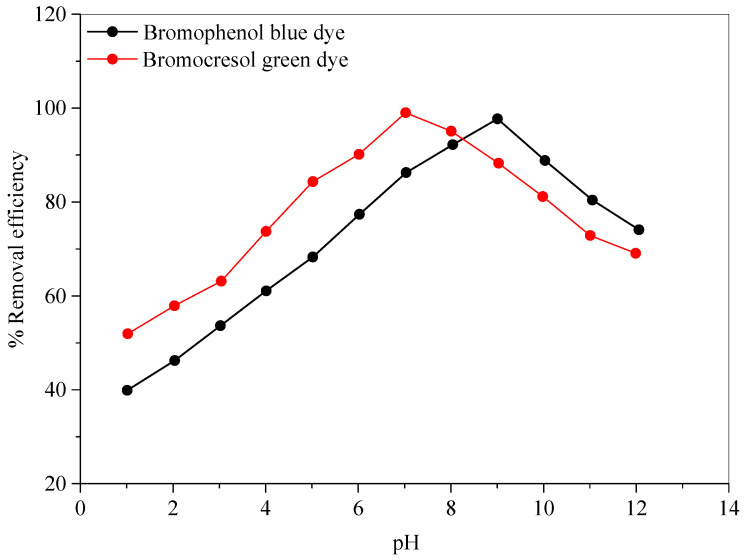
The effect of pH values on the color removal efficiencies of the bromocresol green and bromophenol blue dyes.

The media pH had a significant impact on the decolorization of the dyes. As a result, the effect of pH on the color removal efficiencies for each dye was investigated using (0.1 N) NaOH or (0.1 N) HCl solutions in the pH range of 1–12, under identical conditions for all other parameters, such as Ag-NP dosage, initial dye concentration, and contact time. The results of the effect of pH on the color removal efficiencies for the dyes are presented in Figure 9. This figure demonstrates that color removal efficiency increases from 51.79% to 99.12%, with a maximum value at pH = 7 in the case of the bromocresol green dye. However, in the case of the bromophenol blue dye, the color removal efficiency increases from 40.00% to 97.25%, with the highest value at pH = 9. Hereafter, the removal efficiencies decreased as the pH increased further.

#### 2.2.5. Effect of Stirring Time

**Figure 10:**
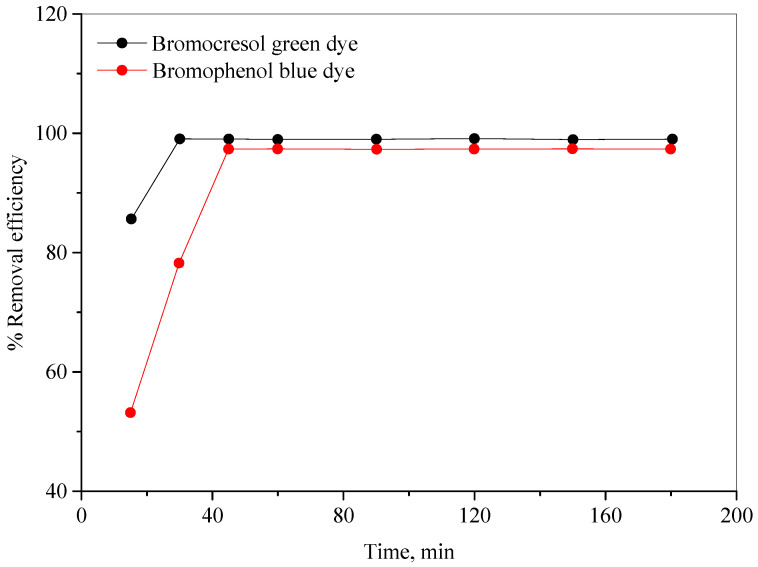
The effect of stirring time on the color removal efficiencies of the bromocresol green and bromophenol blue dyes.

The impact of stirring time on the color removal efficiencies of the BCG and BPB dyes was investigated by testing various times (15–180 min), with all other optimized parameters maintained, and the results are presented in Figure 10. As can be seen in this figure, the stirring time increases with the rise in the color removal efficiencies for the dyes, until it reached its maximum values of 30 and 45 min for the BCG and BPB dyes, respectively. Further extension of stirring time did not result in any noticeable increase in the color removal efficiencies.

#### 2.2.6. Effect of Temperature

**Figure 11:**
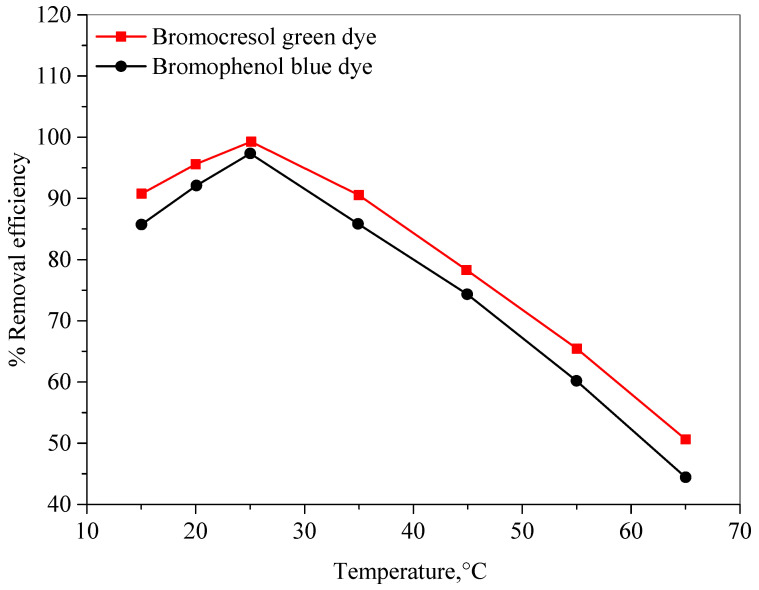
The effect of temperature on the color removal efficiencies of the bromocresol green and bromophenol blue dyes.

Temperature can affect several aspects of adsorption, namely dye solubility, the swelling capacity of the adsorbent, and the equilibrium position associated with exothermic or endothermic from the adsorption phenomenon [46]. The impact of temperature on the color removal efficiencies for the BCG and BPB dyes was studied at temperatures of 15–65 °C, with all optimized parameters maintained, as can be seen in Figure 11. As seen in this figure, the maximum color removal efficiencies were obtained at the room temperature (25 ± 2 °C) for the investigated dyes. Furthermore, raising the temperature above room temperature caused a decrease in the color removal efficiencies. The removal efficiency decreased as the process temperature increased, which is a strong indication that the removal of BCG and BPB dyes by the Ag-NPs is an exothermic process [47].

### 2.3. Reusability and Regeneration of Ag-NPs

**Figure 12:**
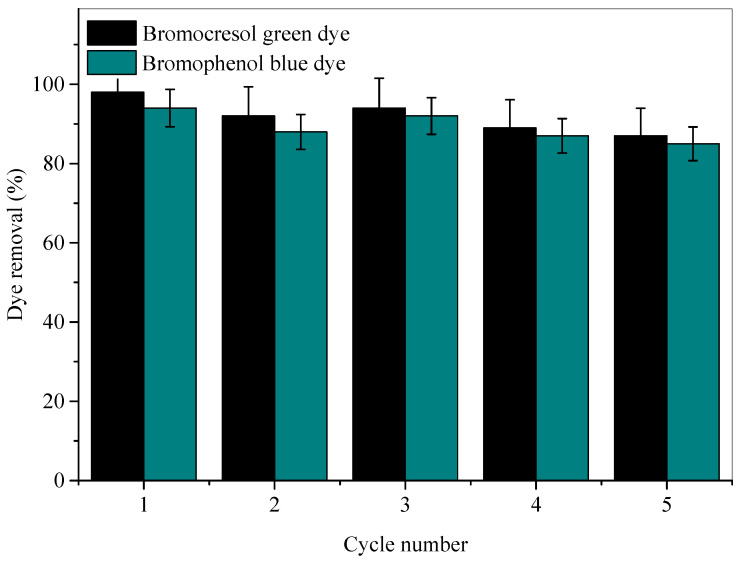
The efficiency of removing BCG and BPB for intact and regenerated Ag-NP adsorbents during five adsorption–desorption cycles.

In order to select the most appropriate adsorbents for application in the commercial sector, it is essential to consider the potential of adsorbents to be recycled or reused. The recycling potential of the Ag-NP adsorbent was investigated via the renewal of the BCG- and BPB-packed adsorbents, which was carried out by swirling the Ag-NPs in ethanol for 5 h, followed by cleaning in ethanol and drying at 50 °C for 5 h. Dye adsorption at optimal renewal conditions for the laden adsorbent was performed five times; the BCG and BPB dye removal rates for five reuses are illustrated in Figure 12. The adsorbents’ adsorption efficiency was somewhat reduced, and although there were several repetitions, the capacity remained significant. This implies that, in addition to being reliable, the Ag-NPs demonstrate excellent recyclability, with data confirming the suitability of their use as adsorbents for water purification through the removal of BCG and BPB dyes.

### 2.4. Adsorption Isotherms

**Figure 13:**
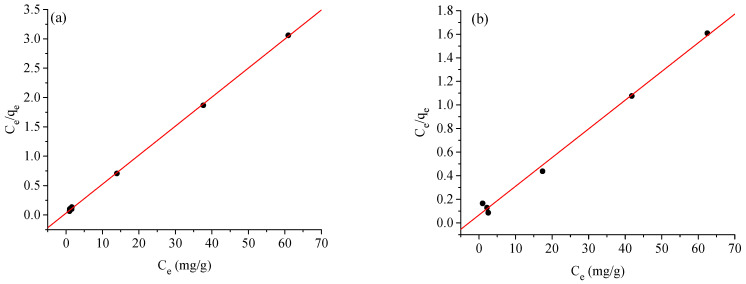

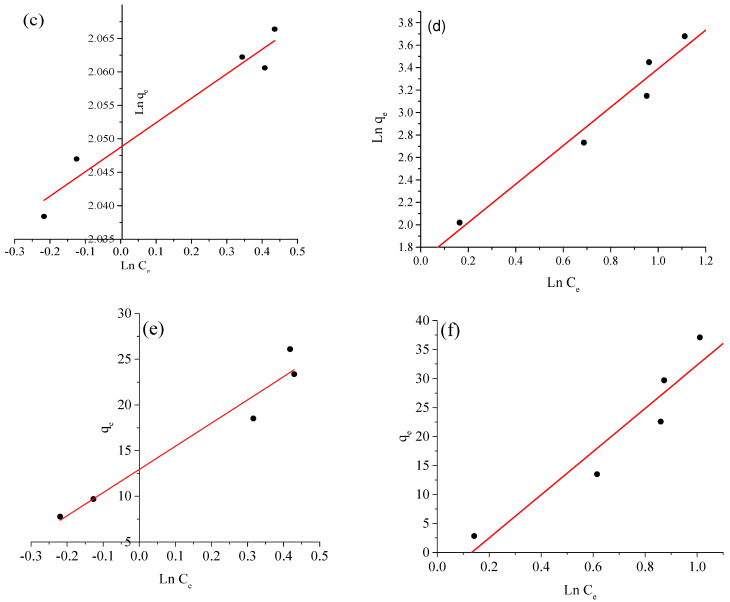
Langmuir (**a**,**b**), Freundlich (**c**,**d**), and Temkin (**e**,**f**) isotherms for the adsorption of bromocresol green (**a**,**c**,**e**) and bromophenol blue (**b**,**d**,**f**) onto the Ag−NP adsorbent.

The Langmuir, Freundlich, and Temkin models were used to evaluate the experimental and theoretical adsorption data for the BCG and BPB dyes. These models were used to classify the Ag-NP adsorbents’ ability to bind to different concentrations of dye with equilibrium under ideal adsorption conditions [48]. According to Langmuir, adsorption occurs equally or simultaneously on the face of an Ag-NP adsorbent. The linear formula of Langmuir may be expressed as follows:

(1) $$\frac{C_{e}}{q_{e}}=\frac{1}{K_{L}q_{m}}+\frac{C_{e}}{q_{m}}\;$$

where C_e_ is the equilibrium concentration of the BCG and BPB dyes in solution (mg L^−1^), q_e_ is the equilibrium adsorption capacity of the BCG and BPB dyes on the Ag-NP adsorbent, q_m_ is the maximum amount of solute that can be adsorbed per gram of adsorbent to form a monolayer (mg g^−1^), and K_L_ is the Langmuir adsorption constant (L mg^−1^). The slope and intercept of the fitted line of C_e_/q_e_ vs. C_e_ (Figure 13a) may be used to compute the variables K_L_ and q_m_. The linear form of the Freundlich (2) isotherm can be given as follows:

(2) $${\mathrm{lnq}}_{e}={\mathrm{lnK}}_{F}+\frac{1}{n}{\mathrm{lnC}}_{e}\;$$

where K_F_ is the Freundlich constant (mg/g), which represents the relative adsorption capacity of the adsorbent; (1/n) is the heterogeneity factor and it is a function of the strength of adsorption process; and n and K_F_ are Freundlich constants related to adsorption intensity and adsorption capacity of the Ag-NP adsorbent, respectively, and they are obtained from the slope and intercept of the linear plot of lnq_e_ against lnC_e_ (Figure 13b).

The Temkin (3) model contains a factor that explicitly takes adsorbent–adsorbate interactions into account. The model assumes that heat of adsorption (function of temperature) of all molecules in the layer would decrease linearly rather than logarithmically with coverage:

(3) $$q_{e}=B_{T}{\mathrm{lnK}}_{T}+B_{T}{\mathrm{lnC}}_{e}\;$$

(4) $$B_{T}=\frac{\mathrm{RT}}{b}$$

A plot of q_e_ versus lnC_e_ enables the determination of the isotherm constants K_T_ and B_T_, as shown in Figure 13c. K_T_ is the equilibrium binding constant (L·mol^−1^) corresponding to the maximum binding energy and constant B_T_ is related to the heat of adsorption. Table 2 lists the estimated adsorption coefficients. R^2^ scores are the main indicators of the precision and caliber of the linear fit procedure [49,50]. The modeling precision is demonstrated by the R^2^ values in Table 2, where Langmuir (R_1_^2^ = 0.9981, 0.9944) > Temkin (R_3_^2^ = 0.8370, 0.8327) > Freundlich (R_2_^2^ = 0.8276, 0.8276) for the BCG and BPB dyes, respectively. Clearly, the adsorption of the BCG and BPB dyes onto the Ag-NP adsorbent follows the Langmuir adsorption isotherm model. This demonstrates that monolayer adsorption takes place in the Ag-NP adsorbent. The calculation results reveal that the maximal single film adsorption capabilities are 20.4081 and 40.7166 mg g^−1^ for the BCG and BPB dyes, respectively. Additionally, fitting the experimental data into a Langmuir isotherm model exhibits the homogeneous nature of the Ag-NP surface, which refers to the adsorption of the BCG and BPB dyes onto the Ag-NP adsorbent as favorable in this investigation at the optimized adsorption conditions.

### 2.5. Adsorption Kinetics

**Figure 14:**
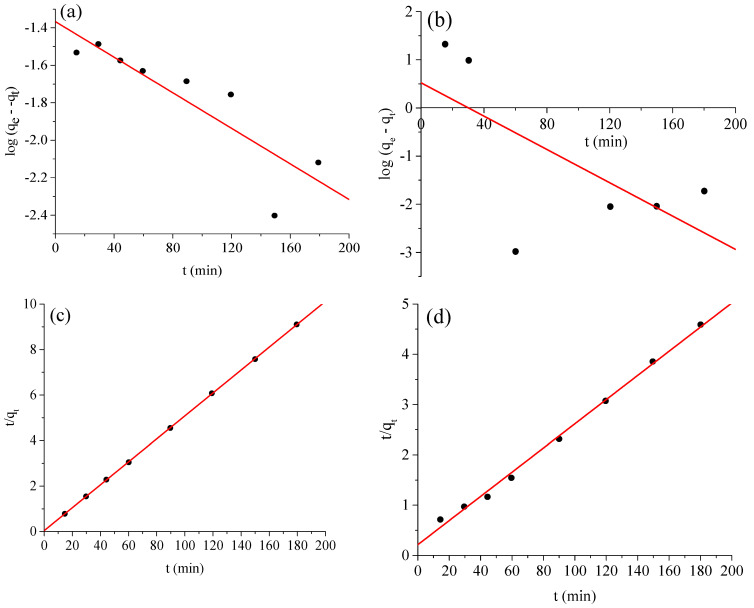
(**a**,**b**) Pseudo-first−order and (**c**,**d**) pseudo−second-order adsorption kinetic models for adsorption of bromocresol green (**a**,**c**) and bromophenol blue (**b**,**d**) onto the Ag−NP adsorbent.

The kinetic variables for the adsorption of the BCG and BPB dyes onto the Ag-NP adsorbent were investigated by employing pseudo-first-order and pseudo-second-order kinetic models, as shown in Figure 14a–d. The aim is to learn more about the quantity of the adsorbent and the rate of the adsorption process. It is critical to note that the magnitude of the linear model, otherwise recognized as the correlation coefficient r^2^, can be used to determine the accuracy and match of the kinetic model. The linear form of the pseudo-first-order kinetic model is as follows:

(5) $$\ln\left(q_{e}-q_{t}\right)={\mathrm{lnq}}_{e}-k_{1}t\;$$

where q_e_ and q_t_ are the BCG and BPB dyes adsorbed (mg g^−1^) at equilibrium and time t (min), respectively. Figure 14a,b show ln(q_e_ − q_t_) against t graphs used to determine the pseudo-first-order adsorption rate constant k_1_ (min^−1^). However, the experimental data do not match the pseudo-first-order kinetic model, and a linear relation could not be derived. Hence, we attempted to fit the adsorption behavior using the pseudo-second-order kinetic model (6), as shown below:

(6) $$\frac{t}{q_{t}}=\frac{1}{k_{2}q_{e}^{2}}+\frac{t}{q_{e}}\;$$

where k_2_ ((g mg^−1^) min) is the pseudo-second-order rate adsorption constant determined by graphing t/q_t_ versus t (Figure 14c,d). Table 2 shows the estimated and observed equilibrium adsorption capacities for the adsorption of the BCG and BPB dyes onto the Ag-NP adsorbent. The pseudo-second-order model can accurately depict the adsorption of BCG and BPB dyes onto Ag-NPs, as shown in Figure 14c,d and Table 3. The predicted and observed adsorption capabilities are identical.

### 2.6. Adsorption Thermodynamics

**Figure 15:**
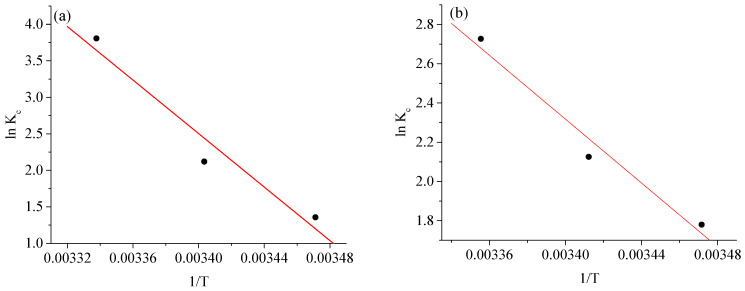
Van ’t Hoff plot for adsorption of (**a**) bromocresol green (BCG) and (**b**) bromophenol blue (BPB) dyes onto Ag-NPs.

The adsorption of BCG and BPB onto Ag-NPs was also assessed using the adsorption thermodynamic concept. In this instance, the Van ’t Hoff graphs were adapted for lnK_c_ vs. 1/T and are depicted in Figure 15a,b. Table 4 lists the pertinent thermodynamic parameters. The ∆G° values were negative in the tested temperature gradient, verifying the spontaneous nature of the thermally advantageous BCG and BPB adsorption processes. The decrease in ∆G° values with temperature shows that higher temperatures favor adsorption. The positive ∆H° values imply the occurrence of endothermic adsorption processes, while the positive ∆S° values indicate random development at the Ag-NP/dye solid–solution interface [49]. Furthermore, the reduction in the ∆G° value of BCG at temperatures ranging from 288 to 298 K demonstrates that its adsorption onto Ag-NPs is promoted by rising temperature. In the case of BPB, the ∆G° value progressively decreases between 288 and 298 K, indicating that the spontaneity of BPB adsorption is not entirely controlled by temperature and that 298 K is a reasonable choice for dye adsorption onto Ag-NPs.

## 3. Experimental Section

### 3.1. Plant Material

In March 2022, aerial parts of *Euphorbia balsamifera* Aiton were gathered from the Asir region along the Khamis–Najran route in Saudi Arabia. The plant was cleaned three times under flowing water, followed by three rounds of dipping in de-ionized water to eliminate any remaining dirt, after which it was allowed to air dry at ambient temperature.

### 3.2. Preparation of Plant Extracts

Aerial parts of *Euphorbia balsamifera* (500 g) were soaked in 90% ethanol (2.0 L) for 72 h at 25 °C. The ethanol extract was then filtered, and the extraction process was performed three times. The bulk extract was concentrated after being defatted with n-hexane in order to create the primitive extract (4.0 g) before spray-drying with hot air.

### 3.3. Green Synthesis of Silver Nanoparticles (Ag-NPs)

**Figure 16:**
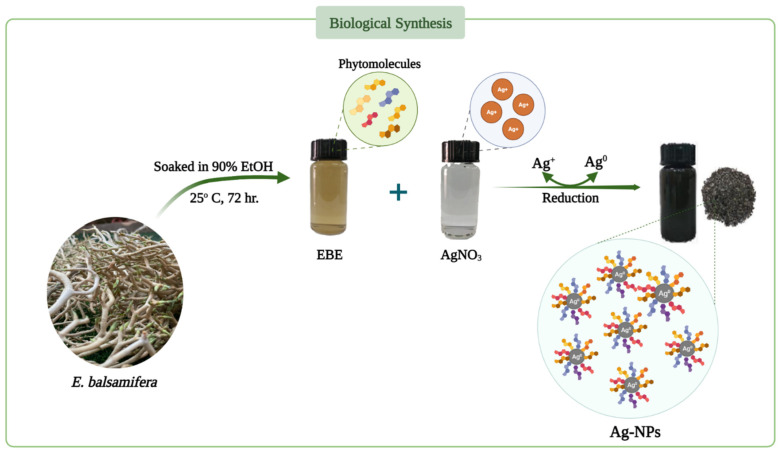
Schematic diagram for the biosynthesis of Ag-NPs using extracts of the aerial parts of *E. balsamifera*.

AgNO_3_ (0.017 g) in 100 mL of distilled water was added to the *Euphorbia balsamifera* extract (EBE) for digestion. Various ratios (1:1, 1:2, 1:3, 1:4, *v*/*v*) of AgNO_3_ and EBE were continuously agitated for 30 min in the dark chamber and intermittently heated (45 ± 5 °C) until a unique hue was attained following the reduction in silver ions caused by bioactive components, indicating the formation of Ag-NPs. Centrifugation at 10,000 rpm for 20 min resulted in the collection of synthesized Ag-NPs, which were washed thrice with distilled water to remove traces of any unbound phyto-constituents. The generated Ag-NPs exhibited the maximum absorbency when mixed at a 1:1 ratio. Thermal drying of the material produced solid Ag-NPs that were used in the following studies (Figure 16).

### 3.4. Characterization

The FT-IR 460 PLUS Spectrophotometer was used to record the IR spectrum in KBr discs in the 4000–400 cm^−1^ range, with a 4 cm^−1^ resolution, and an average of 16 FTIR scans were collected for each spectrum. The BET (Brunauer–Emmett–Teller) surface area was calculated using Quantachrome TouchWin™ (version 1.2 Copyright ©1994–2015) with nitrogen adsorption–desorption measurements. The crystallinity was determined at ambient temperature using an X-ray diffractometer (XRD, ADX2500, Stoughton, MA, USA). A field emission scanning electron microscope (FE-SEM) connected to a microscope was used to capture FE-SEM images (JEOL JSM-6500F). The material was disseminated in ethanol on a copper grid, and transmission electron microscopy (TEM) images were captured using a JEM 2100 electron microscope at an accelerating voltage of 200 kV. The T80 UV/Vis dual spectrometer (PG Instruments Ltd., Lutterworth, UK) was used to take measurements of samples in 10.0 mm-fitted quartzite cells in the spectral range of 2.0 nm. The Adwa pH-meter (Model AD 1030, Romania) and the Digital Hotplate stirrer (Model MSH-20D, made by DAIHAN Scientific Co., Ltd., Seoul, Republic of Korea) were also used, as well as the Centrifuge PLC series (Model PLC-03, USA) with a power of 220 V/50 HZ; 0.65 A.

### 3.5. Reagents

AR-grade bromocresol green dye (C_21_H_14_Br_4_O_5_S), bromophenol blue dye (C_19_H_10_Br_4_O_5_S), hydrochloric acid (HCl, 37%), silver nitrate (AgNO_3_, 99.99%), and sodium hydroxide (NaOH, 99.95%) were acquired from Merck Ltd. in Germany and utilized without additional purification. All aqueous solutions were prepared in de-ionized water.

### 3.6. Preparation of Dye Solutions

Stock solutions comprising 500 mg/L of each dye (bromocresol green and/or bromophenol blue, Table 5) were prepared by adding about 0.25 g of each dye into a 500 mL volumetric flask with double-distilled water added up to mark. A series of concentrations of each dye, ranging from 1.0 to 150.0 mg/L, were prepared through dilution.

### 3.7. General Procedure for Decolorization of Dyes

A total of 25 mL of each dye solution (bromocresol green (BCG) and bromophenol blue (BPB)), with an initial concentration of 100 mg/L in a 100 mL calibrated flask, was mixed with 60 and 80 mg Ag-NPs. The pH values of the solutions were adjusted to pH = 7 for BCG dye and pH = 8 for BPB dye using (0.1 N) NaOH. The BCG dye mixture was stirred for 30 min in a flask at room temperature, while that of BPB was stirred for 45 min. Finally, the samples were centrifuged at 6000 rpm for 10 min, and the residual concentration of each dye was analyzed by UV/Vis spectrophotometry; the calibration curves were prepared from measurement of the BCG and BPB dyes at 615 and 590 nm, respectively [24,25,26].

The color removal efficiency was calculated for each dye using the following equation:

$$\%\;\mathrm{Color}\;\mathrm{removal}=\frac{C_{i}-{\;C}_{f}}{C_{i}}\times 100$$

where C_i_ and C_f_ are, respectively, the initial and final concentrations (mg/L) of the dye.

## 4. Conclusions

In conclusion, Ag-NPs were successfully synthesized using *Euphorbia balsamifera* plant extract as a potent bioresource, thus representing a simple, cost-effective, and ecological approach for the synthesis of Ag-NPs. This study highlights the effective application of nanotechnology in water treatment. The results of XRD, IR, UV, SEM, TEM, EDAX, BET, and thermal analyses demonstrated the efficient fabrication of Ag-NPs and revealed their spherical shape and small size distribution. The synthesized Ag-NPs exhibited an excellent ability to adsorb the bromocresol green (BCG) and bromophenol blue (BPB) dyes. At pH = 7, 60 mg of Ag-NPs absorbed approximately 99.12% of the 100 mg/L BCG dye in 30 min, while at pH = 9, 80 mg of Ag-NPs absorbed approximately 97.25% within 45 min, for the adsorption of both dyes at room temperature. The adsorption kinetics and thermodynamics were also studied. We conclude that the adsorption process studied here is simple, effective, and low-cost and may be utilized for the decolorization of water containing BCG and BPB dyes.

## Figures and Tables

**Table 1:** The average crystallite size of Ag-NPs was estimated using the XRD pattern.

No of Peaks	2θ (Degree)	θ	FWHM	Height	d Value [Å]	(hkl)	t
1	38.05	19.07	0.4351	456.4	2	111	19.12
2	44.32	22.16	0.5428	143.9	2	200	15.64
3	64.70	32.26	0.4872	94.3	1	220	19.09
4	77.35	38.68	1.0103	94.4	1	311	09.97

**Table 2:** Variables of the isotherm models for the adsorption of the bromocresol green (BCG) and bromophenol blue (BPB) dyes onto the Ag-NP adsorbent.

Isotherm Models	Variables	Value	
		BCG	BPB
Langmuir	K_L_ (L mg^−1^)	01.2800	00.5329
	q_m(cal)_ (mg g^−1^)	20.4081	40.7166
	R_1_^2^	00.9981	00.9944
	R_L_	00.0077	00.0184
Freundlich	K_F_ (mg g^−1^) (L mg^−1^)^1/n^	07.7566	5.3924
	R_2_^2^	00.8276	00.8276
	n	28.8350	00.5384
Temkin	B_T_ (J. mol^−1^)	25.2973	33.1412
	K_T_ (L g^−1^)	01.6521	00.9929
	R_3_^2^	00.8370	00.8327

**Table 3:** Kinetic constants for the adsorption of bromocresol green (BCG) and bromophenol blue (BPB) dyes onto the Ag-NP adsorbent.

Kinetic Models	Variables	Value	
		BCG	BPB
Pseudo first order	k_1_ (min^−1^)	9 × 10^−5^	0.0003
	q_e(cal)_ (mg g^−1^)	39.644	38.900
	r_1_^2^	0.7753	0.4335
	q_e(exp)_ (mg g^−1^)	0.2832	1.468
Pseudo second order	k_2_ [g mg^−1^ min^−1^]	1.600	0.0033
	q_e(cal)_ (mg g^−1^)	39.644	38.900
	r_2_^2^	1	0.9959
	q_e(exp)_ (mg g^−1^)	39.821	41.0340

**Table 4:** Thermodynamic parameters for the adsorption of bromocresol green (BCG) and bromophenol blue (BPB) dyes onto Ag-NPs.

Temperature (K)	$\ln K_{c}$		$\Delta G^{{}^{\circ}}\left(kJ/mol\right)$		$\Delta H^{{}^{\circ}}\left(kJ/mol\right)$		$\Delta S^{{}^{\circ}}\left(kJ/mol\right)$	
	BCG	BPB	BCG	BPB	BCG	BPB	BCG	BPB
288	1.3707	0.845	−3.282	−2.023	173.503	128.528	0.6125	0.4526
293	2.1248	1.508	−5.176	−0.004				
298	3.8078	2.649	−9.434	−0.006				

**Table 5:** Characterization and chemical structures of the bromocresol green (BCG) and bromophenol blue (BPB) dyes.

	Bromocresol Green, (BCG)	Bromophenol Blue, (BPB)
Dye IUPAC name	3,3-Bis(3,5-dibromo-4-hydroxy-2-methylphenyl)-3*H*-benzo [c] [1,2]oxathiole 1,1-dioxide	3,3-Bis(3,5-dibromo-4-hydroxyphenyl)-2,1-benzoxathiole-1,1(3*H*)-dione
Chemical structure	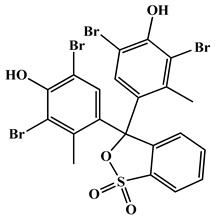	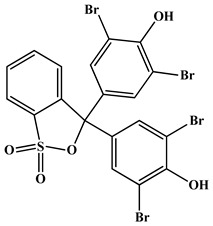
Molecular formula	C_21_H_14_Br_4_O_5_S	C_19_H_10_Br_4_O_5_S
Chemical class	Sulfonephthaleins	Phenolphthaleins
λ_max_	600 nm	590 nm
Type	Anionic dye	Anionic dye
Solubility	Soluble in NaOH, water (6 mg/mL), ethanol (40 mg/mL), benzene, and diethyl ether.	Soluble in NaOH, methyl and ethyl alcohols, benzene, and acetic acid. Slightly soluble in water (0.4 g/100 g) at 20 °C.
CAS Number	Bromocresol green 76-60-8	Bromophenol blue 115-39-9

## Data Availability

The datasets used and/or analyzed during the current study are available from the corresponding authors upon reasonable request.
